# Correlating the morphology of *Anacardium occidentale* L. fruits from 30 orchards with their physicochemical and nutritional properties

**DOI:** 10.3389/fpls.2022.1033577

**Published:** 2022-12-01

**Authors:** Roger Dakuyo, Kiessoun Konaté, David Bazié, Abdoudramane Sanou, Kabakdé Kaboré, Hemayoro Sama, Balmoussa Santara, Frédéric Anderson Konkobo, Mamoudou Hama Dicko

**Affiliations:** ^1^ Laboratory of Biochemistry, Biotechnology, Food Technology and Nutrition (LABIOTAN), Department of Biochemistry and Microbiology, University Joseph, Ouagadougou, Burkina Faso; ^2^ Applied Sciences and Technologies Training and Research Unit, Department of Biochemistry and Microbiology, University of Dedougou, Dedougou, Burkina Faso; ^3^ Training and Research Unit in Life and Earth Sciences, Nazi BONI University, Bobo-Dioulasso, Burkina Faso

**Keywords:** cashew fruit, morphological traits, nutritional properties, antinutritional factor, *anacardium occidentale*

## Abstract

Cashew (*Anacardium occidentale* L.) is a cross-pollinating plant whose fruit consists of two parts, the nut, and the apple. This study aimed to carry out agro-morphological characteristics of cashew fruits to establish relationships with their physicochemical and nutritional properties. Thirty (30) cashew accessions fruits were sampled in different regions of Burkina Faso. The length, width, thickness, weight, yield, kernel output ratio (KOR), proximate composition, specific minerals, phenolic compounds, and anti-nutritional factors were assessed. Evaluations were made using standard methods. The results showed significant variations in agro-morphological, physicochemical and nutritional traits depending on the accessions and the organ. Also, the ratio of apple mass versus nut one was about 12.24 ± 1.24. Kernels are an important source of proteins, fat, total phenolic compounds, and flavonoids, with average contents of 22.84 ± 1.25 g/100 g, 51.65 ± 2.54 g/100 g, 9.78 ± 2.15 mg GAE/g, and 6.24 ± 12.15 mg QE/g, respectively. As for the apples, they contained substantial quantities of Potassium, Phosphorus, Chlorine, and Magnesium with contents of 611.24 ± 14.5 mg/100 g, 418.24 ± 16.47 mg/100 g, 332.24 ± 10.54 mg/100 g, and 224.95 ± 13.15 mg/100 g, respectively. Statistical analyses showed that mineral and phytate contents were positively correlated to cashew apples while phenolic compounds and tannins were strongly correlated with walnuts. Principal component analyses showed three groups of accessions based on apples and kernels characteristics. These data showed a direct relationship between cashew physicochemical, nutritional potentials, kernels, and apples’ agro-morphological characteristics. These data constitute an important basis for the identification of cashew accessions with high nutritional and economic potential.

## Introduction

Cashew (*Anacardium occidentale L*.) belongs to the family Anacardiaceae, characterized by tropical and subtropical trees and shrubs. There are more than sixty genera and four hundred species related to the genus of *Anacardium* (Borges, 2021). The word “Anacardium” is of Greek origin and means “inverted heart” in allusion to the shape of the fruit (Oliveira et al., 2020). Cashew trees have a diversity of characteristics ranging from large Amazonian trees, reaching up to 40 m in height and with a sparsely developed fleshy pseudo-fruit or cashew apple (*Anacardium giganteum*), to medium-sized arboreal forms, not exceeding 4 m in height (*Anacardium othonianum*), to trees not exceeding 80 cm in height (*Anacardium humile)* (Abdul and Peter, 2010). *Anacardium occidentale* L. is a plant with considerable economic, nutritional and medicinal value (da Silva et al., 2018). It is native to the Caribbean and Northeast Brazil (Liaotrakoon et al., 2016; Havik et al., 2018; Duangjan et al., 2021). It is currently grown in many tropical countries around the world and its production is expanding every year due to the importance of fruits in food industries.

The cashew tree is adapted to the humid tropical climate. It is an allogamous plant whose fruit displays two parts: the nut, which is the true fruit in the botanical sense of the term, and the apple, which is called the false fruit. It is edible and used as nutrient and medicinal food (Oliveira et al., 2020). The apple generally consumed by local populations is juicy and often processed into juice (Akinwale, 2000; Prasertsri, 2017). The nut is hard and consists of the shell, the kernel, and the cashew nut shell liquid (CNSL). The kernel is the most prized part of the nut because it is rich in fat and proteins (Guedes-Oliveira et al., 2016). The kernel is used to make peanuts, and sometimes as a substitute for milk powder in chocolate making (Semiu Olalekan Ogunwolu et al., 2009), while the shell is often used to produce energy through pyrolysis (Godjo et al., 2015). CNSL is highly acidic, corrosive, rich in phenolic compounds, and is used for several applications in the chemical industry (Andrade et al., 2011; Sharma et al., 2020). Nuts contain many bioactive compounds with antioxidants and anti-inflammatory properties (Trevisan et al., 2006; Andrade et al., 2011). The apple is juicy and is consumed by local populations. However, for storage difficulties and because of its astringent taste, its consumption is sometimes limited in certain regions. This restrained consumption is also explained by certain prejudices such as its incompatibility with milk (Emmanuelle et al., 2016). The apple color ranged from yellow to red because of its richness in pigments (Adegunwa et al., 2020). Because of its nut, the cashew tree is one of the most produced nut plants in the world, mainly in Africa, Vietnam, India, Cambodia, Brazil, and Indonesia (Sisconeto Bisinotto et al., 2021). It is a plant with multiple virtues whose therapeutic and nutritional importance of the leaves, barks, roots, nut, apple, and their derivatives has been demonstrated (Hamad and Mubofu, 2015; Salehi et al., 2019). Several studies have also shown the nutritional importance of nuts, as well as their economic contribution (Havik et al., 2018; Semporé et al., 2021b). Studies in some regions have shown that apples and cashew kernels have high nutritional potential (Salehi et al., 2019). Cashew nuts contain protein contents of 25.3 ± 0.2 g/100 g (Aremu et al., 2006) and that the most representative high amino acids were: glutamic acid (22.4-13.6%), aspartic acid (5.6-10.2%) and leucine (6.2-8.0%) (Venkatachalan and Sathe, 2006). The high quality of the cashew nut’s amino acid intake is also confirmed by the high value of essential amino acids, representing up to 47% of the total (Aremu et al., 2007). It has been proven that apples contain huge amounts of ascorbic acid reaching 218.933 ± 5.331 mg/100g and 279.37 mg.100 g^-1^ respectively by (Singh et al., 2019) and (de Lopes et al., 2012). The nectar of the cashew fruit has interesting antifungal properties (Oliveira et al., 2020) and antioxidant activities because of its richness in bioactive compounds (Andrade et al., 2011). They also contain many types of pigments such as chlorophyll, anthocyanins, and carotenoids including beta-carotene (provitamin A) (Schweiggert et al., 2016). Cashew apples contain anthocyanin contents of about 21.16 mg.100 g^-1^ (de Lopes et al., 2012) and 20.79 mg.100 g^-1^ of vitamin A (Assunção and Mercadante, 2003). Some studies have focused on their agro-morphology characteristics of nuts (Semporé et al., 2021a). However, very little data exists on the agro-morphological traits of apples. Moreover, no study has shown the link between agro-morphological traits and the nutritional properties of cashew fruits. Thus, these data are essential for the selection and promotion of accessions or cultivars producing fruits of better nutritional quality. This study focuses on characterization of agro-morphological, physicochemical, and nutritional potential of apples and kernels of Cashew accessions grown in three regions of Burkina Faso in order to established links between *Anacardium occidentale* L. fruit morphological traits and their nutritional and functional constituents. Principal Component Analyses (PCA) were performed to classify the production sites according to their nutritional composition and specially to highlight the existing correlations between the nutritional parameters and the apples and cashew nuts.

## Material and methods

### Plant material and sample collection

**Figure 1:**
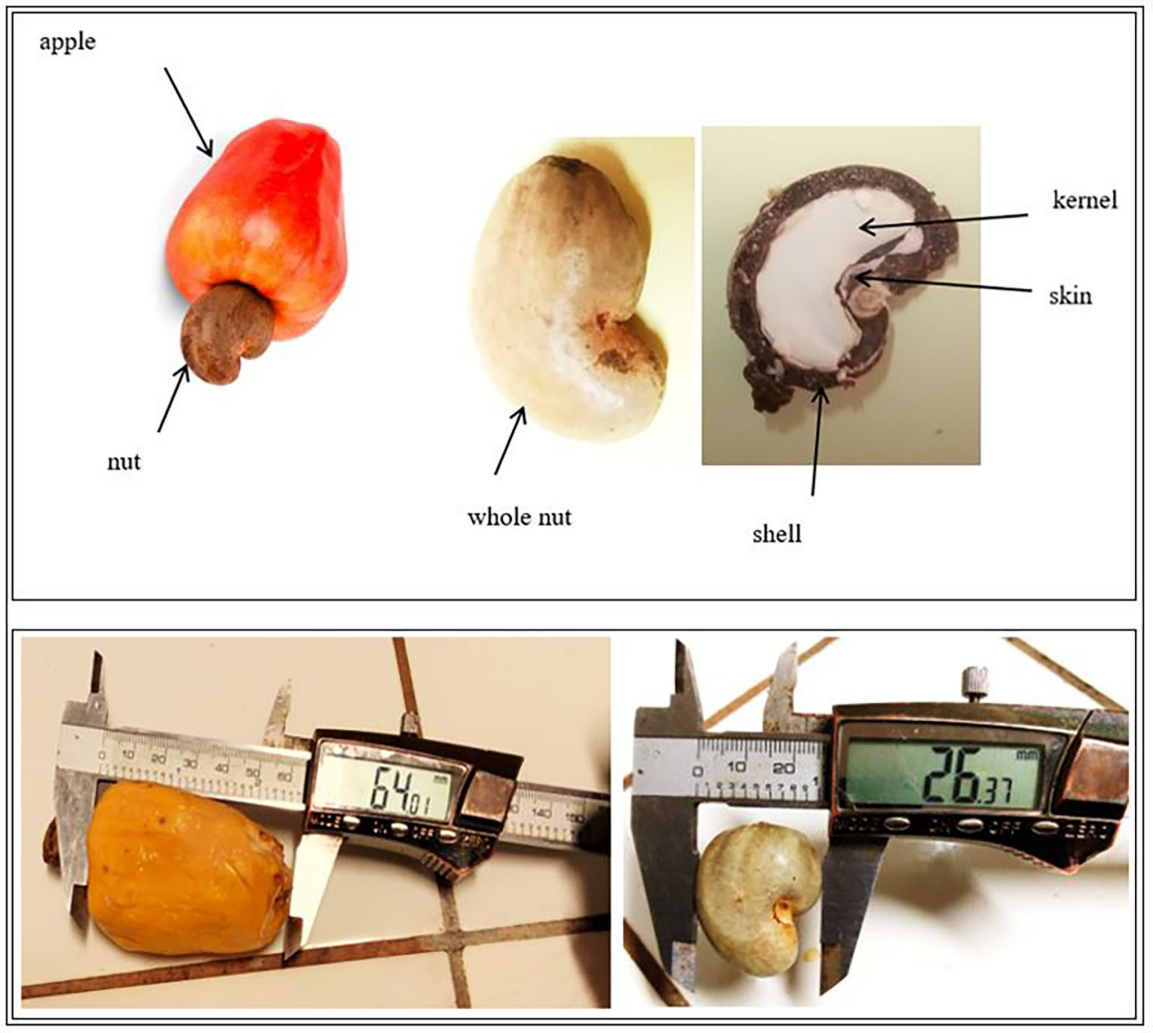
Parts of the cashew fruit (apple and nuts).

Cashew, *Anacardium occidentale* L. (**Figure 1**) fruits were collected from 30 orchards in Burkina Faso (13 00 N, 2 00 W). These orchards were selected based on their high production in last five years. Fruits were collected in the three largest producing regions of Burkina Faso, i.e., the South-West region in Gaoua (10° 17’ 57” N, 3° 15’ 3” W), the Haut-Bassins region in Bobo-Dioulasso (11° 10’ 37.7” N, 4° 17’ 52.4” W) and the Cascades region in Banfora (10° 37’ 60” N, 4° 46’ 0” W). These three (03) regions are all subject to a tropical climate of the South Sudanese type, marked by two seasons: the wet season from April to October and the dry season from November to March. Rainfall is relatively good. It ranges from 800 to 1200 mm, 900 to 1200 mm, and 100 to 1200 mm respectively for the Haut-Bassins, South-West, and Cascades regions. The Haut-Bassins and South-West regions have ferruginous and hydromorphic soils while the soils of the Cascades region are ferruginous, hydromorphic, and lithosols. The cashew trees in the concerned orchards were between 7 and 21 years old with an average age of 10.5 years.

**Figure 2:**
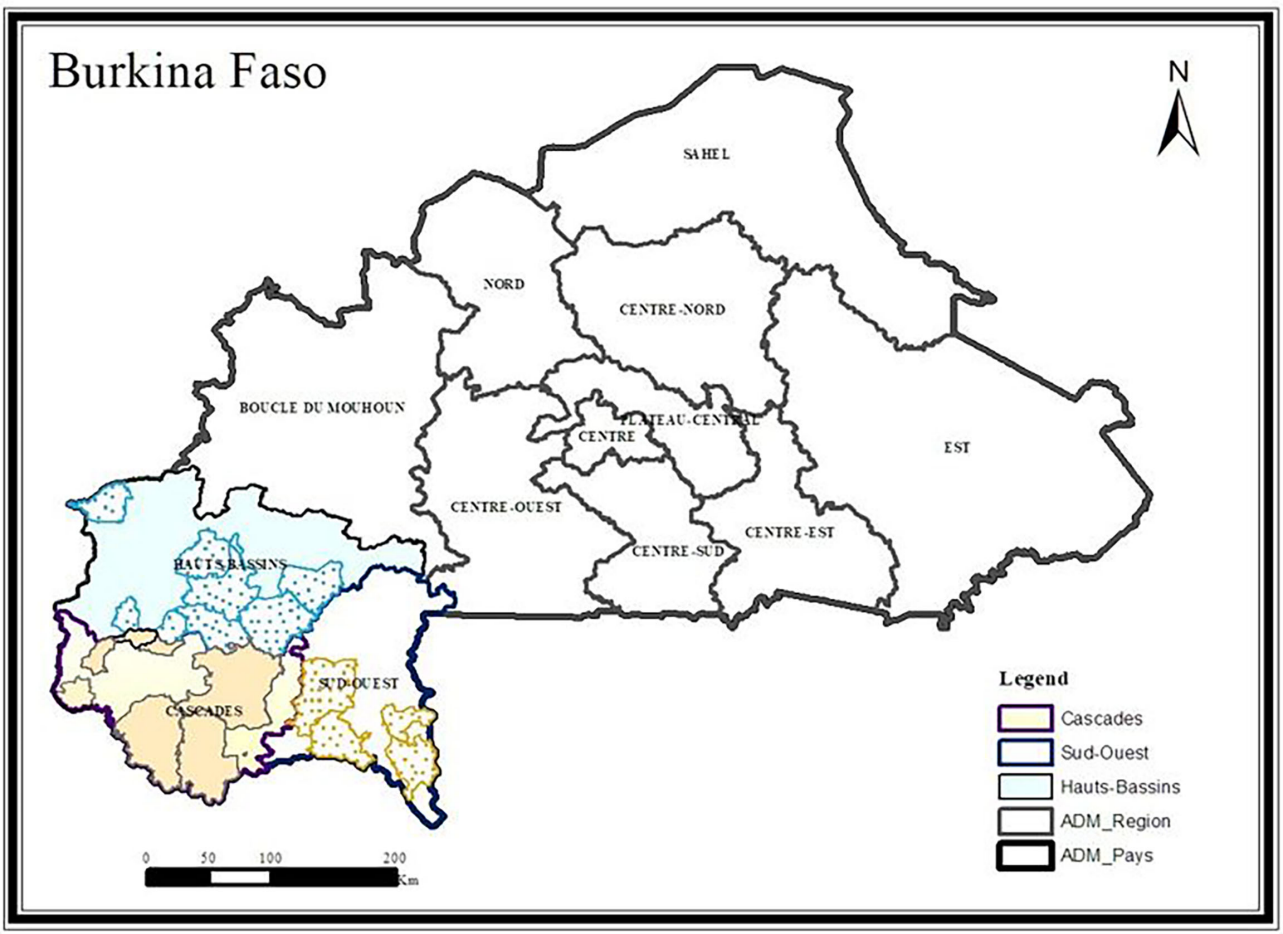
Sample map showing collection sites in the three regions.

In each orchard, samples of cashew nuts and apples were collected. For sample collection, each orchard plot was subdivided into four subplots along the diagonals. In each subplot, 1000 g of fresh apples and 500 g of cashew nuts were collected. The apples were picked directly from the cashew trees or under them if they were firm. The nuts were harvested under the cashew trees, where they were left for up to 5 days, depending on the producer. The apples were packed in coolers at 4°C to avoid spoilage and transported to the laboratory for analyses. Nuts put conserved in fiber bags prior to conservation. In each region, 10 orchards were selected for collection (**Table 1**). The sampling locations are shown on the map above (**Figure 2**).

**Table 1:** Collection areas of cashew cultivars by region and their code.

Regions	Hauts-Bassins	Cascades	Sud-Ouest
**Harvesting site**	Toussiana (S_1_)	Sideradougou (S_11_)	Kampti (S_21_)
	Péni (S_2_)	Serefedougou (S_12_)	Loropeni (S_22_)
	Yeguéresso (S_3_)	Beregadougou (S_13_)	Batié (S_23_)
	Bama (S_4_)	Kouere (S_14_)	Tonkar (S_24_)
	Orodora (S_5_)	Moussodougou (S_15_)	Doudou (S_25_)
	Santidougou (S_6_)	Fourkoura (S_16_)	Iolonioro (S_26_)
	Koumbia (S_7_)	Sindou (S_17_)	Koronkoura (S_27_)
	Niamberla (S_8_)	Foloni (S_18_)	Dioro (S_28_)
	Samandeni (S_9_)	Mangodara (S_19_)	Dipéo (S_29_)
	Karangasso-Vigué (S_10_)	Niangoloko (S_20_)	Kparanta (S_30_)

### Samples preparation

For some dosages, cashew nuts were shelled and skinned manually using small secateurs. The kernels obtained were ground and then degreased by extraction with n-hexane using the Soxhlet apparatus. The resulting flour was used as a matrix for some analyses.

In some cases, the apples were carefully cleaned and then dried in a ventilated dryer (Meller 110, Prolab dryer) at 30°C until a constant weight was obtained.

### Agro-morphological characteristics determination

The agro-morphological parameters taken into account in this study are mainly length, width, thickness, weight, kernel yield, KOR (kernel output ratio), and seediness for cashew nuts. For cashew apples, determined agro-morphological parameters were length, width, thickness, and weight.

The three main components of the dimensions of the nuts and apples, namely length, width, and thickness, were determined by measuring with a caliper (Castorama LR44, Germany) with an accuracy of 0.01 mm. For each type of sample, 10 apples and 10 nuts were randomly selected and measured in order to calculate the average dimensions for samples as previously described (Li et al., 2015). Cashew fruit weight was determined using an analytical balance with an accuracy of 0.0001 g (RADWAG AS 220.R2, Poland). For each orchard, 10 cashew nuts and 10 cashew apples were randomly selected and weighed five times to calculate the average weight per collection site.

The kernel yield is the most important indicator of the quality of raw nuts. It allows estimating from a sample, the average quality of useful kernels of a production. The nuts have been shelled and the kernels separated from the shells and the skin. The kernel yield (KY) was calculated using the following equation (1) by Ref. (Ogunwolu et al., 2016):


(1) $$KY=\frac{{\text{m}}_{2}+\left(\frac{{\text{m}}_{3}}{2}\right)}{{\text{m}}_{1}}*100$$

Where: m_1_: total mass of the nut sample

m_2_: mass of healthy kernels (kernels + pellicle) accepted at 100%

m_3_: mass of kernels + pellicle of the nuts rejected at 50%.

The kernel output ratio (KOR) was defined as the amount in pounds (1 Lbs = 0.45359 kg) of good kernel per bag of 80 kg of raw nuts after shelling. It is expressed in Lbs/bag of 80 kg (**Table 2**). The KOR was calculated using the following Equation (2) as reported by:


(2) $$\text{KOR}=\frac{\text{KY}}{100}*\frac{80}{0.43359}$$

**Table 2:** KOR evaluation grid for kernels.

KOR category (lbs)	Appreciation
<180 nuts	Very large nuts, sought after quality
[180–190[	Large nuts, appreciated by industrialists, good KOR
[190–200[	Large nuts, appreciated by processors
[200–210[	Medium nuts, most common in West Africa Medium
[210–220[	Medium nuts, more common in West Africa
[220–230[	Small nuts, not much in demand Small
[220–230[	Small nuts, difficult to process

The number of nuts or graining was defined as the number of nuts per kg and reflects the average nut size. Therefore, the assessment of the graining quality was determined using the grid (**Table 3**) proposed by (Rongead, 2015).

**Table 3:** Graining quality assessment grid.

Amplitude classes	Quality assessment	Description of nuts Very
<180 nuts	Excellent	Very large nuts, sought after quality
[180–190[	Very good	Large nuts, appreciated by industrialists, good KOR
[190–200[	Good	Large nuts, appreciated by processors
[200–210[	Medium	Medium nuts, most common in West Africa Medium
[210–220[	Very average	Medium nuts, more common in West Africa
[220–230[	Juste acceptable	Small nuts, not much in demand Small
[220–230[	Poor	Small nuts, difficult to process

### Physicochemical properties

For moisture content, fruit samples were weighed and dried in an oven at 105°C for 3 h. Afterwards the weights of sample before and after drying were used to calculate the moisture content (AOAC, 2000).

For determination of ash, samples were weighed in triplicates into a pre-weighed crucible. The crucibles along with samples were placed in a muffle furnace at 550°C overnight until white ash was obtained. The samples were taken out the next day and placed in the desiccator.

### Proximate composition

Crude proteins were determined by the Bradford method (Bradford, 1976) with slight modifications. (Bolek et al., 2016). Samples (500 mg) of apple and almond flours were homogenized in 10 mL of 0.1 M NaCl, and the whole was stirred for 5 h at 150 rpm/min at 25°C. The extract was collected after centrifugation at 4400 rpm for 30 min at 4°C. To 50 μL of each extract were added 250 μL of Bradford reagent. After incubation for 2 min, absorbances are read at 595 nm. A standard curve was built using BSA as standard.

Cabohydrate were determined by the phenol-sulfuric acid method with few modifications (Chow and Landhäusser, 2004). Glucose was used as standard allowing to build a linear plot.

The total fat content of the samples was determined by gravimetrical method according to the standard of AOAC 2003.05 (2012) using a Soxhlet apparatus (R040605, Gerhardt, Germany). Fat content was calculated using equation (3):


(3) $$\text{Fat content}(\%\text{DM})=\frac{\text{W}1-Wo}{Ws}\;\times 100$$

Where, W_0_: Weight of the empty balloon (g); W_1_: Weight of the balloon after extraction and drying (g); and Ws: Initial sample weight (g).

Potential energy value was estimated using the Atwater coefficients. The calorific value of the sample is calculated (Daugherty and Watkins, 1976) as follow:


(4) Energy value = P x 4 Kcal + G x 4 Kcal + L x 9 Kcal= X kcal /100 g

where: P, G, L are the proportions of proteins, carbohydrates, and lipids, respectively.

Starch content was determined according to the spectrometric method (Jarvis and Walker, 1993). Rice starch was used as a standard, allowing to obtain a linear curve.

### Phenolic compounds

Total phenolic compounds were quantified according to the procedure described by Singleton et al. (1999) modified by Hasperué et al. (2016). It is based on the high oxidizability of phenolic compounds. The colorimetric properties of the Folin-Ciocalteu Reagent (FCR) are modified when it is complexed with certain molecules. It reacts with the OH function of phenols. The absorbances are read at 760 nm with a spectrophotometer. Gallic acid is used as a reference compound.

The content of total flavonoids was performed by the colorimetric method described by Arvouet-Grand et al. (1994). This method is based on the aluminum chloride (AlCl_3_) test. 0.5 ml of methanol of each fraction extract solution (0.1 mg/ml) was mixed with 1.5 ml of AlCl_3_ (2%) and incubated for 30 min at room temperature after this incubation period. The absorbance was read spectrophotometrically against a blank at 415 nm. The results were expressed as mg quercetin equivalent (QE) per gram of fresh material.

### Anti-nutritional factors

The contents of hydrolysable tannins were quantified according to the method of Price & Butler (1977) with slight modifications. Briefly, 1 mL of extract (5 mg/mL) was added to 3.5 mL of a solution prepared from 0.01 M ferric trichloride (FeCl_3_) in 0.001 M hydrochloric acid (HCl). After 15 seconds, the absorbance of the mixture was read at 660 nm. The results were expressed as mg ascorbic acid equivalent (GAE) per g dry extract (mg GAE/100 g). The hydrolysable tannins were determined by the following formula (5):


(5) $$Hydrolysable\;tannins(\%)=\frac{A\;\times \;MW\;\times \;V\;\times \;DF}{\epsilon \;\times \;m}$$

A: absorbance; M_W_: molecular weight of tannic acid (170.12 g/mol);

V: volume of extract used; DF: dilution factor; ϵ (molar extinction of gallic acid): 2169 M^-1^ cm^-1^); m: mass of the sample in g.

Phytate extraction was performed by mixing 250 mg of flour in 10 mL of 2.4% HCl for 3 h at room temperature with constant stirring. The samples were clarified by centrifugation at 6 000 rpm for 20 min at room temperature (Gonçalves et al., 2020). The supernatant was applied and eluted from an anion-exchange resin (Dowex1x8-400, Sigma Co.). The assay was performed with 2.0 mL of Wade reagent [0.03% (w/v) FeCl_3_ and 0.3% sulfosalicylic acid] and 3.0 mL of the eluted sample. The phytate content was determined at 500 nm using a spectrophotometer using phytic acid as standard (Latta and Eskin, 1980).

### Minerals content

Minerals: Zn, Fe, Mg, Mg, Mn, P, Ca, Na and Cl were quantified by atomic absorption spectroscopy (AAS), PerkinElmer, Waltham, MA, ICP- OES (inductively coupled plasma optical emission spectroscopy, Varian Inc./Agilent Technologies) and ICP-MS (inductively coupled plasma mass spectroscopy, Agilent Technologies). The method is based on that of (Borowiak et al., 2014). The content of the samples was determined using a calibration curve [0 - 50µg] for each measured element.

### Statistical analysis

Graphs and the different concentrations calculations were done using GraphPad Prism version 8.4.3, and Excel 2016. Data were subjected to the analyses of variance (ANOVA) and significant differences between means were revealed *via* the Tukey test (p< 0.05) and was done using XLSTAT (2016) software. Principal component analysis and dendrogram were performed using R, version 4.0.2 (2020) software.

## Results and discussion

### Agro-morphological characterization

The fruits of *Anacardium occidentale* L. have different forms. It should be noted that they were nuts which are called “true fruits” and apples which are called “false fruits”. The agro-morphological parameters used to characterize cashew nuts were thickness, length, width, weight, kernel yield, KOR, and seediness. For apple thickness, length and weight were considered (**Table 4**). Cashew nut dimensions showed variations in length, thickness, and width. The thickness of the cashew nuts varied between 14.91 ± 1.06 cm and 18.86 ± 0.68 cm. The lengths varied from 27.19 ± 0.213 cm to 36.21 ± 1.073 cm, while the widths of the nuts were between 21.856 ± 0.182 cm and 26.26 ± 0.327 cm. As for the dimensions of the cashew apple, the values are higher than those of the nuts, which is quite logical because the apple at simple sight is more voluminous than the nut. Thus, the largest thickness was 50.053 ± 1.455 cm observed for apples at S_5_ and the smallest thickness was 36.873 ± 1.5 in S_11_. The lengths of the apples were more remarkable they were between 46.253 ± 1.402 cm and 81.843 ± 2.468 cm for S28 and S_30_ respectively. As for the width of the apples, it varied between 24.953 ± 2.311cm and 54.153 ± 1.335 cm. Our results are similar to previous studies (Semporé et al., 2021a). The different values of the dimensions allowed to observe various shapes. For example, oval-shaped apples whose length, width, and thickness are very close; to elongated-shaped apples whose length is sufficiently greater than the width and thickness. Physical characteristics such as the size of agricultural products are important for the design of sorting and storage equipment, material handling in various mechanical systems, and various packaging and processing machines (Abdel-Sattar et al., 2021). The measurement of dimensions are parameters that that indicate the volume of the fruits of the various localities and they allow to make varietal selections according to the characteristics of sought-after forms. The selection of fruit plants such as cashew is generally based on agro-morphological characteristics, productivity and nut-eating quality (Semporé et al., 2021a).

**Table 4:** Agromorphological characteristics of cashew apples and nuts.

	Cashew nuts							Cashew apples				
	Thickness (cm)	Length (mm)	Width (mm)	Weight (g)	Yield (%)	KOR	Graining	Thickness (mm)	Length (mm)	Width (mm)	Weight (g)	Wa/Wn ratio
S1	18.86 ± 0.68^a^	29.76 ± 0.74^b^	24.93 ± 1.78^b^	5.95 ± 0.4^b^	27.14 ± 0.93^ab^	47.87 ± 1.64^b^	185.45 ± 4.5^b^	49.78 ± 1.75^a^	51.72 ± 1.18^c^	49.46 ± 0.82^a^	75.15 ± 2.45^ab^	12.63
S2	17.05 ± 0.46^ab^	32.76 ± 2.38^ab^	25.31 ± 1.74^b^	5.74 ± 0.5^b^	25.67 ± 0.65^b^	45.27 ± 1.12^bc^	192.45 ± 3.2^ab^	42.21 ± 2.51^b^	55.75 ± 2.99^bc^	37.46 ± 1.72^b^	64.24 ± 4.24^b^	11.19
S3	16.63 ± 1.94^ab^	33.07 ± 2.15^a^	25.33 ± 1.16^b^	8.5 ± 1.0^a^	28.47 ± 1.60^ab^	50.22 ± 2.86^ab^	152.45 ± 2.4 ^ab^	45.66 ± 2.04^ab^	58.47 ± 1.95^bc^	38.61 ± 81b^b^	70.24 ± 34^ab^	8.26
S4	16.77 ± 0.51^ab^	31.36 ± 2.19^ab^	25.04 ± 1.98^b^	5.24 ± 1.5 ^ab^	24.09 ± 2.59^b^	42.49 ± 4.53^c^	200.2 ± 7.47^a^	46.90 ± 2.29^ab^	57.38 ± 2.12^bc^	45.81 ± 2.26^ab^	86.12 ± 4.5^a^	16.44
S5	18.59 ± 0.69^ab^	30.91 ± 0.84^ab^	25.19 ± 0.84^b^	5.83 ± 0.75^b^	25.42 ± 0.84^b^	44.84 ± 1.45^bc^	191.5 ± 2.8^ab^	50.03 ± 1.55^a^	60.97 ± 1.68^b^	46.53 ± 1.47^ab^	88.54 ± 3.75^a^	15.19
S6	16.42 ± 1.83^ab^	32.33 ± 1.09^ab^	24.76 ± 0.62^b^	6.25 ± 1.1^ab^	25.92 ± 2.30^b^	45.73 ± 4.92^bc^	186.45 ± 5.47^b^	46.08 ± 2246^ab^	50.13 ± 1.79^c^	40.33 ± 1.87^b^	79.15 ± 4.52^ab^	12.77
S7	17.81 ± 1.40^a^	28.64 ± 0.8^bc^	22.35 ± 1.22^c^	5.91 ± 0.7^b^	27.10 ± 3.39^b^	47.80 ± 5.97^b^	192.5 ± 6.4^ab^	44.12 ± 1.31^b^	61.49 ± 1.49^b^	37.98 ± 1.8^b^	84.45 ± 3.44^a^	14.29
S8	16.73 ± 1.07^ab^	31.26 ± 1.89^b^	24.13 ± 1.87^bc^	7.29 ± 0.75^ab^	28.09 ± 1.38^ab^	49.55 ± 2.48^ab^	175.45 ± 3.47^b^	46.45 ± 1.68^ab^	66.45 ± 1.33^ab^	41.66 ± 174^b^	83.15 ± 6.15^a^	11.27
S9	17.60 ± 0.52^a^	31.45 ± 0.53^b^	24.59 ± 1.29^b^	6.29 ± 0553^ab^	27.61 ± 1.42^ab^	48.71 ± 2.58^b^	182.45 ± 5.3^b^	46.33 ± 1.22^ab^	51.4 ± 2.06^c^	41.76 ± 2.25^b^	75.15 ± 2.45^ab^	11.95
S10	17.16 ± 0.47^ab^	32.88 ± 2.14^ab^	26.94 ± 0.86^a^	5.79 ± 0.78^b^	26.64 ± 1.27^ab^	46.99 ± 2.23^b^	195.45 ± 4.75^b^	46.50 ± 2.67^ab^	60.40 ± 1.58^b^	40.43 ± 1.41^b^	76.15 ± 6.15^ab^	13.15
S11	17.73 ± 1.26^a^	31.21 ± 1.56^b^	25.92 ± 0.46^ab^	5.4 ± 0.59^b^	24.50 ± 1.96^b^	43.21 ± 3.49^c^	205.45 ± 3.5^a^	36.87 ± 1.57^b^	55.36 ± 1.93^bc^	24.53 ± .11^c^	64.15 ± 4.72^b^	11.88
S12	16.69 ± 0.36^ab^	36.21 ± 1.73^a^	27.35 ± 0.18^a^	7.11 ± 1.2^ab^	26.01 ± 0.89^b^	45.87 ± 1.53^bc^	184.5 ± 6.4^b^	40.83 ± 2.29^b^	52.28 ± 2.54^c^	35.03 ± 1.04^bc^	69.34 ± 3.6^b^	9.75
S13	14.91 ± 1.06^b^	31.86 ± 0.98^ab^	24.21 ± 0.23^b^	5.92 ± 0.72^b^	24.88 ± 3.31^b^	43.88 ± 5.88^c^	194.5 ± 5.6^ab^	40.32 ± 1.72^b^	50.07 ± 1.55^c^	43.36 ± 2.11^b^	73.15 ± 4.57^ab^	12.36
S14	16.94 ± 0.75^ab^	30.96 ± 0.39^b^	23.33 ± 1.35^bc^	5.5 ± 0.95^b^	25.42 ± 0.68^b^	44.83 ± 1.17^bc^	190.1 ± 4.5^ab^	39.03 ± 2.09^b^	64.50 ± 1.46^ab^	40.66 ± 1.58^b^	81.45 ± 3.15^ab^	14.81
S15	18.33 ± 1.45^a^	30.56 ± 1.22^b^	24.80 ± 1.62^b^	6.05 ± 1.12^b^	26.09 ± 0.32^ab^	46.01 ± 0.59^bc^	187.5 ± 3.54^b^	46.08 ± 2.18^ab^	61.77 ± 0.88^ab^	41.76 ± 2.14^b^	75.6 ± 4.85^ab^	12.50
S16	16.91 ± 0.49^ab^	31.73 ± 1.19^ab^	23.97 ± 0.26^b^	6.15 ± 0.7^b^	26.91 ± 0.92^ab^	47.46 ± 1.69^b^	187.45 ± 7.62^b^	39.6 ± 1.72^b^	53.22 ± 2.01^b^	42.03 ± 1.68^b^	73.23 ± 3.4^b^	11.91
S17	17.40 ± 0.28 ^ab^	29.06 ± 0.42^bc^	23.52 ± 0.93^bc^	8.2 ± 1.2^a^	28.81 ± 0.26^a^	50.81 ± 4.72^ab^	175.45 ± 3.21^b^	46.08 ± 2.38^ab^	59.95 ± 1.44^b^	44.86 ± 2.25^ab^	78.6 ± 8.48^b^	9.59
S18	16.3 ± 0.36^ab^	27.19 ± 0.23^c^	21.85 ± 0.82^c^	5.25 ± 1.11^b^	23.98 ± 1.10^b^	42.30 ± 1.49^c^	211.45 ± 6.47^a^	43.12 ± 2.76^b^	53.21 ± 2.12^c^	41.64 ± 0.76^b^	70.8 ± 4.67^b^	13.49
S19	16.38 ± 0.57^ab^	31.52 ± 0.93^b^	24.08 ± 1.32^b^	5.32 ± 1.04^b^	25.83 ± 0.23^b^	45.56 ± 0.4^bc^	197.5 ± 4.5^ab^	43.73 ± 2.45^b^	52.36 ± 2.59^c^	41.09 ± 1.79^b^	72.15 ± 2.15^ab^	13.56
S20	18.02 ± 1.23^a^	31.43 ± 1.96^b^	26.26 ± 0.37^a^	8.22 ± 0.65^a^	29.07 ± 0.91^a^	51.27 ± 1.07^ab^	165.42 ± 3.2^bc^	45.36 ± 0.78^ab^	59.97 ± 1.65^bc^	45.76 ± 0.35^ab^	76.15 ± 2.15^ab^	9.26
S21	15.25 ± 0.45^b^	30.33 ± 1.26^b^	24.26 ± 0.44^b^	5.55 ± 0.75^b^	25.99 ± 0.53^b^	45.84 ± 0.38^bc^	200.2 ± 5.8^a^	49.12 ± 2.217^b^	63.35 ± 0.63^b^	54.13 ± 1.33^a^	78.34 ± 6.32^ab^	14.12
S22	15.56 ± 1.89^b^	29.66 ± 1.36^b^	23.89 ± 0.82^b^	6.01 ± 0.84^b^	24.49 ± 1.07^b^	43.20 ± 1.89^c^	198.5 ± 7.5^a^	42.09 ± 1.28^b^	60.57 ± 1.62^b^	48.73 ± 3.22^a^	80.24 ± 4.5^ab^	13.35
S23	18.71 ± 1.44^a^	30.76 ± 0.44^b^	25.66 ± 0.16^bc^	6.25 ± 0.25^ab^	26.59 ± 1.12^ab^	46.89 ± 1.87^b^	185.15 ± 4.3^ab^	42.4 ± 2.53^b^	56.50 ± 1.08^bc^	39.15 ± 0.56^b^	74.67 ± 52^b^	11.95
S24	16.62 ± 1.82^ab^	29.86 ± 0.69^b^	25.24 ± 3.56^ab^	8.08 ± 0.8^ab^	27.85 ± 0.84^ab^	49.11 ± 1.87^b^	175.64 ± 3.4^b^	49.06 ± 2.56^a^	63.58 ± 1.02^b^	40.23 ± 0.70^b^	83.54 ± 4.21^a^	10.34
S25	17.51 ± 2.72^a^	31.16 ± 2.32^b^	25.11 ± 1.48^b^	8.72 ± 0.5^a^	27.58 ± 2.16^ab^	48.65 ± 3.13^b^	168.42 ± 6.54^bc^	44.07 ± 2.65^a^	61.97 ± 1.71^b^	40.73 ± 1.76^b^	76.84 ± 3.4 ^ab^	8.81
S26	16.17 ± 0.33^ab^	32.02 ± 0.92^ab^	24.78 ± 0.93^b^	9.02 ± 0.7^a^	30.61 ± 0.71^a^	54.13 ± 1.68^a^	154.45 ± 5.45^c^	49.7 ± 1.63^a^	64.49 ± 1.36^ab^	44.53 ± 1.27^ab^	87.24 ± 2.45^a^	9.67
S27	16.83 ± 0.83^ab^	29.95 ± 0.88^b^	23.49 ± 0.5^bc^	5.83 ± 0.47^b^	26.52 ± 1.77^ab^	46.30 ± 3.12^b^	192.45 ± 6.3^ab^	48.26 ± 3.25^ab^	58.09 ± 2.04^bc^	45.62 ± 2.31^ab^	76.48 ± 4.10^ab^	13.12
S28	17.37 ± 0.84^ab^	31.02 ± 0.77^b^	24.65 ± 1.27^b^	6.07 ± 0.95^b^	27.61 ± 1.76^ab^	48.80 ± 3.15^b^	187.64 ± 4.57^ab^	40.75 ± 2.33^a^	46.25 ± 1.40^c^	45.40 ± 1.50^ab^	72.36 ± 5.2^ab^	11.91
S29	15.89 ± 0.88^b^	30.37 ± 2.46^b^	23.81 ± 0.72^b^	7.96 ± 0.8^ab^	28.33 ± 0.94^ab^	49.97 ± 1.62 ^ab^	178.15 ± 4.5^b^	48.88 ± 0.88^ab^	78.17 ± 3.01^a^	46.35 ± 1.48^ab^	88.49 ± 2.15^a^	11.12
S30	17.69 ± 1.64^a^	31.73 ± 0.67^b^	25.23 ± 1.38^b^	8.95 ± 0.57^a^	29.43 ± 1.02^a^	51.91 ± 1.02^ab^	152.14 ± 4.1^c^	52.58 ± 2.44^a^	81.84 ± 2.46^a^	43.35 ± 3.26^b^	89.24 ± 3.42^a^	9.97

Superscript letters denote the statistical differences (results of the ANOVA analysis) between the samples for the parameters considered.

S means “sample collection site” and the numbers 1 to 30 indicate the number assigned to each site as listed in **Table 1**.

It appears that apples weighed more than the nuts to which they are coupled (**Table 4**). The mass of apples ranged from 64.15 ± 4.72 g for S_11_ to 89.24 ± 3.42 for S_30_, whereas the mass of nuts was from 5.24 ± 1.5 (S4) to 9.02 ± 0.7 g (S_26_). The ratio of mass of apples versus nuts (Wa/Wn) varied between 8.81 and 16.44 among samples. The determination of the weight ratio of the apple versus the nut is very important because it can help in the varietal selection of accessions that produce large and heavy nuts when we know the economic importance of cashew nuts. Kernel yield and KOR are very dependent agronomic and commercial data. The lowest yield and KOR were observed for accession S_18_ and were respectively 23.986 ± 1.105% and 42.304 ± 1.949 Lbs while the most interesting yield and KOR were observed for accession 30.691 ± 0.719% and 54.131 ± 1.268 Lbs. Graininess which represents the number of nuts per Kg of nut is an important parameter to appreciate the quality of cashew nuts and the productivity of cashew trees. These values oscillated from 152.14 ± 4.1 and 200.2 ± 5.8 nuts/Kg. In this case, according to the scale established by (Rongead, 2015) (**Table 4**), 30% of the nuts have excellent graining, 30% have very good graining, 26.66% have good graining, 10% have average graining and 3.33% have low graining. The cashew nut has a high market value due to its interesting biochemical composition. Thus, the agronomic parameters that act as quality criteria for the nuts greatly influence the commercial balance at the time of sale (Ndiaye et al., 2020). That is why these findings are important for cashew products, to identify varieties with important fruit characteristics. These different data are therefore of capital importance for producers and traders. According to the RONGAED assessment grid, the nuts as a whole present interesting agronomic characteristic because they have a good seed content and especially an excellent KOR. This should allow producers to make substantial savings and return on their investment in production (Havik et al., 2018). These findings corroborate the previous ones on experimental cashew accessions (Semporé et al., 2021a). The almond yields of our accessions are higher than those (Ndiaye et al., 2020) which found average yields between a, 23.57% and 28.34% in a study conducted in Senegal.

### Physico-chemical and proximate composition

**Figure 3:**
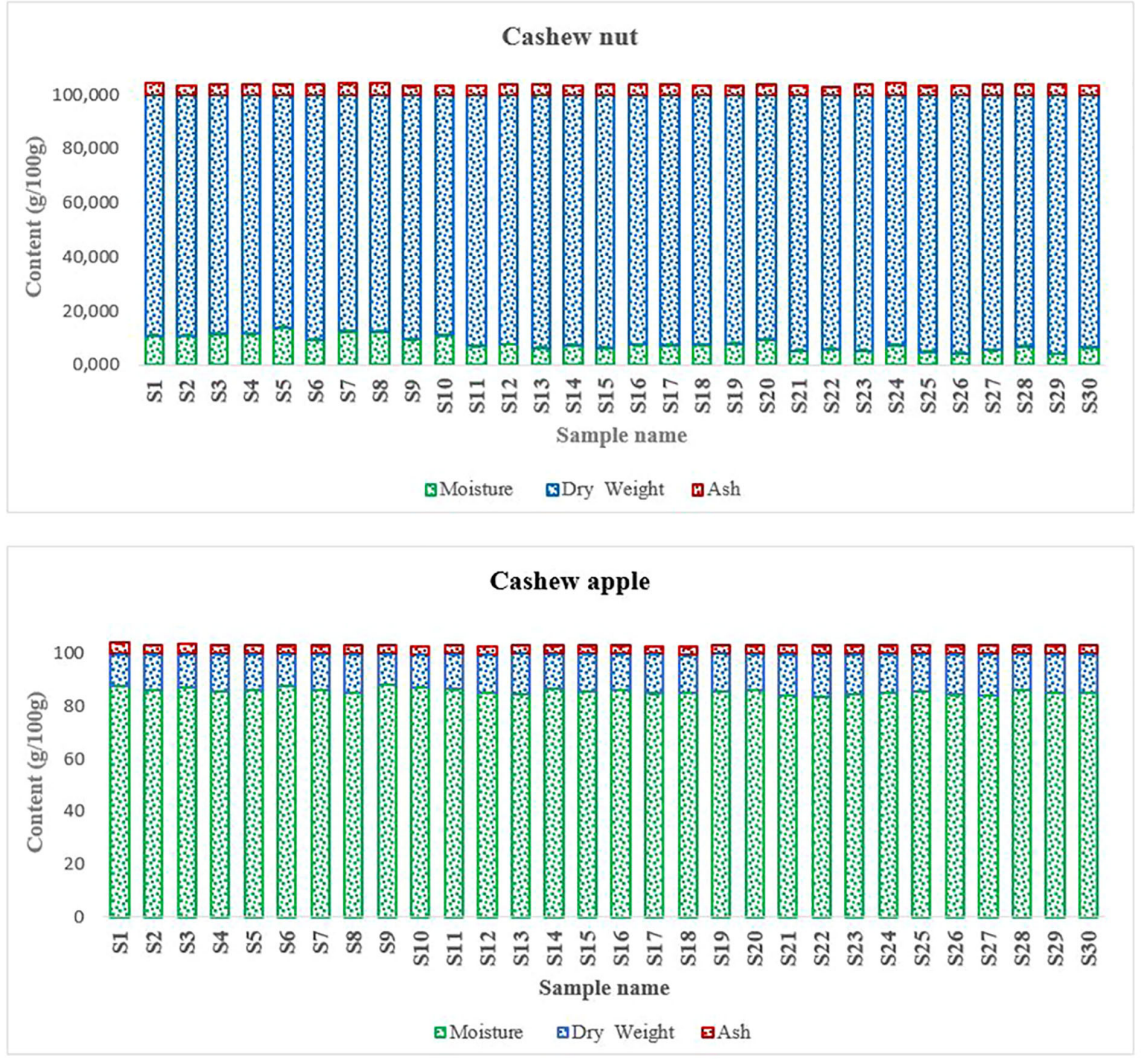
Physical properties of cashew fruits.

The two components of the fruit showed different physicochemical characteristics (**Figure 3**). The moisture content is essential data, especially for products to be marketed such as cashew nuts. Thus, the apple is generally consumed as a fleshy fruit but also mainly processed into juice and even sometimes into wine (Lowor and Agyeute-Badu, 2009; Leite et al., 2021; Adou et al., 2014). Its high water content poses conservation problem (Singh et al., 2019; Sá et al., 2020). On the other hand, the eastern walnut has a long shelf life, but it can be also altered during storage. During storage, it can lose some of its fat and especially the cashew nut shell liquid (CNSL) which is quite acidic (Koteich Khatib et al., 2020).

**Figure 4:**
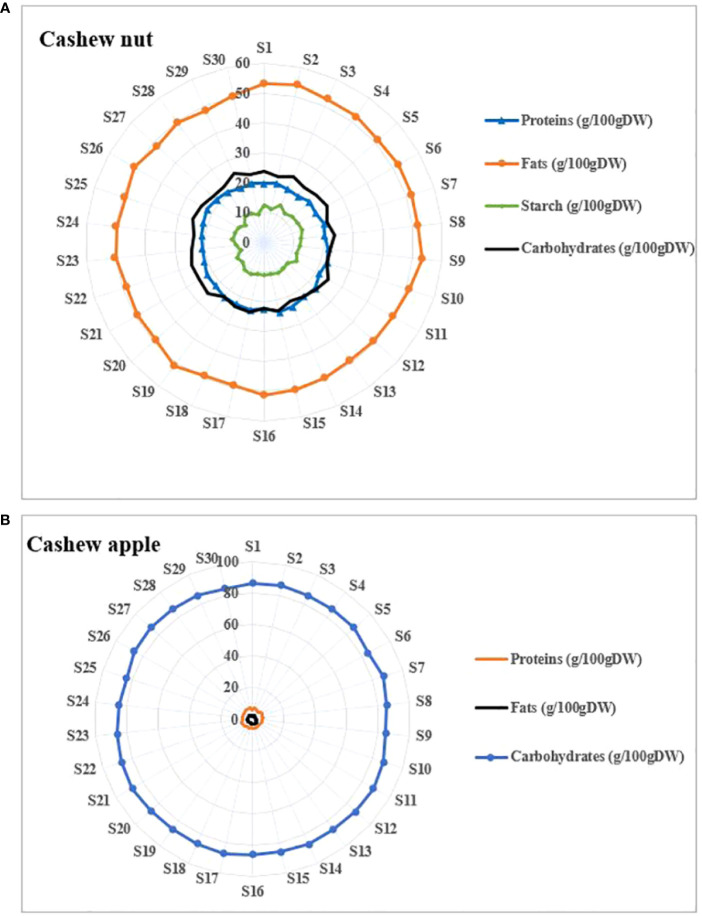
Proximate composition of cashew fruits. **(A)** apples, **(B)** nuts.

Cashew apples are safe bet in terms of nutrition and diet and they contain very important nutrients (Bai et al., 2019). Apples are sweet (Akinwale, 2000) and naturally consumed in their raw state. Apples contained about 86 g/100 g Dry Weight (DW) (**Figure 4**). This high carbohydrate content partly explains some characteristics of cashew apple juice such as their sweetness (Adegunwa et al., 2020). In addition, the literature indicates that cashew apples are among the richest fruits in vitamin C (Akinwale, 2000). Studies have shown that cashew apple juice contains special pectins, oligosaccharides, glucans and tagatose that play essential roles in the intestinal flora (Tamiello-Rosa et al., 2019; Leite et al., 2021). As for nuts they contain medium levels of carbohydrates compared to apples. The total carbohydrate contents were around 23.94 ± 2.24 g/100 g DW (**Figure 4**). These contents are slightly higher than those found by Rico et al. (2016) and Semporé et al. (2021b) who found carbohydrate contents of the order of 20.5 g/100 g and 21.18 ± 3.81%, respectively. Cashew nuts contain free oses such as galactose, arabinose, glucose, rhamnose, mannose, and xylose that intervene in several metabolic processes in the organism (de Oliveira Silva Ribeiro et al., 2020). Kernels contain significant amounts of starch of about 10.45 g/100 g, which gives them quite high swelling capacity.

Very low protein contents are observed in apples compared to nuts. Indeed, the lowest protein content in apples was 5.52 g/100 g DW fsor S1 and the highest content was 7.12 g/100 g DW for S_22_ (**Figure 4**). The protein contents of nuts ranged from 19.67 g/100 g DW and 24.02 g/100 g DW. Our results are similar to previous studies such as Rico et al. (2016); Semporé et al. (2021b) who found protein contents of 21.18 ± 3.81 g/100 g and 21.3 g/100 g in cashew kernels, respectively. Protein in cashew kernels has important techno-functional properties (Liu et al., 2018). High protein content (34%, w/w) was found in defatted cashew kernel flours (Emelike et al., 2015). There are more and more interest using cashew kernels rich in protein in formulation of food products (Fong-in et al., 2020; Lima et al., 2021).

The fat is the major component of the cashew nut. Indeed, kernels contained high level in fat (48.27 and 54.25g/100 g DW) while apples contained only traces of it (1.63 and 3.52g/100 g DW). These data are close to those found elsewhere (Rico et al., 2016). This level of fat in cashew kernels confers potential uses in the manufacturing process of butter, cheese, peanut, etc. Cashew kernels, therefore, have an oilseed potential comparable to other known oilseeds such as cottonseeds (Bolek et al., 2016) but lower than peanuts and palm nuts (Atasie et al., 2009). Cashew kernel oil is interesting for human health because it is rich in unsaturated as well as omega-3 fatty acids (*e.g*. linoleic acid) liposoluble vitamins such as vitamin E (Rico et al., 2016; Koné et al., 2020).

### Phenolic compounds, antinutritional factors and minerals content

**Figure 5:**
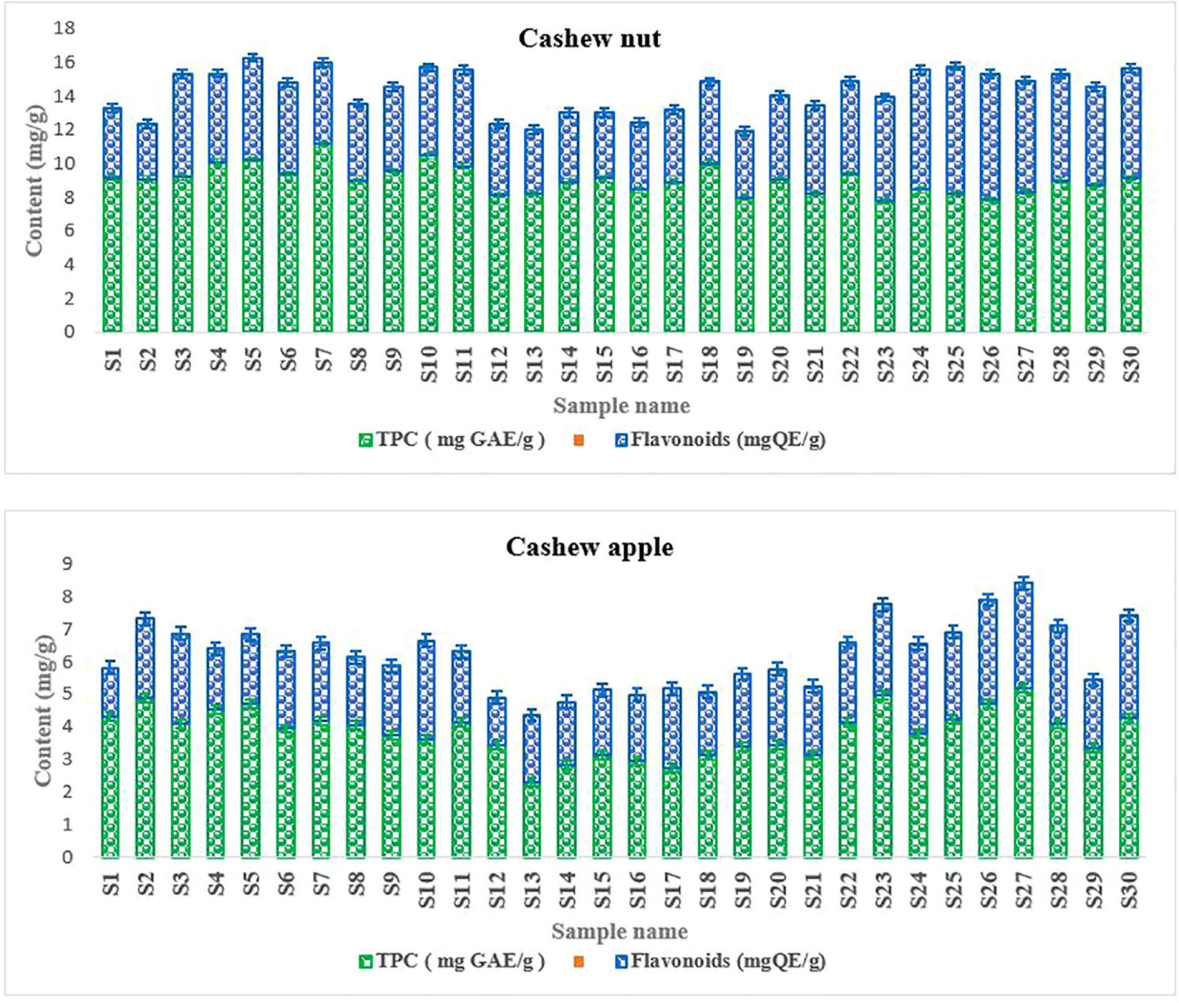
Phenolic compounds content.

Phenolic compounds are secondary metabolites that are well known for their biological properties such as antioxidant, antimicrobial, and antiviral capabilities and many other biological properties. Phenolic compounds present in fruits and vegetables have attracted much interest due to their antioxidant potential (Alda et al., 2009). Polyphenol levels ranged from 7.8 to 11.2 mg GAE/g for walnuts and from 2.7 to 5.01 mg GAE/g for apples (**Figure 5**). For flavonoids, levels ranged from 3.6 to 7.54 mg GAE/g for walnuts and from 1.7 to 5.21 mg GAE/g for apples (**Figure 5**). Indeed, the presence of phenolic compounds in the different parts of the plants constitutes a natural defense against pests (Cortés-Estrada et al., 2020). In addition, they confer antioxidant and anti-inflammatory properties to the different parts of the plant. In addition, these compounds may contain active principles of interest to treat certain diseases, especially to prevent metabolic diseases (Gonçalves et al., 2020). Thus, they reduce oxidative stress, which contributes to reducing the risk of metabolic diseases that cause many deaths in recent years. For the specific case of flavonoids, it should be noted that this class of secondary metabolites have antioxidant properties capable of scavenging free radicals, thus protecting cells from oxidative stress (Kibiti and Afolayan, 2015; Moriasi et al., 2020). Moreover, it appears that flavonoids have antimicrobial, antiviral, anti-allergic, and anti-aging properties and are very important for animal and human health because of their antioxidant and antitumor properties (González-Vallinas et al., 2015). All these data show the nutraceutical importance of the fruits of cashew.

**Figure 6:**
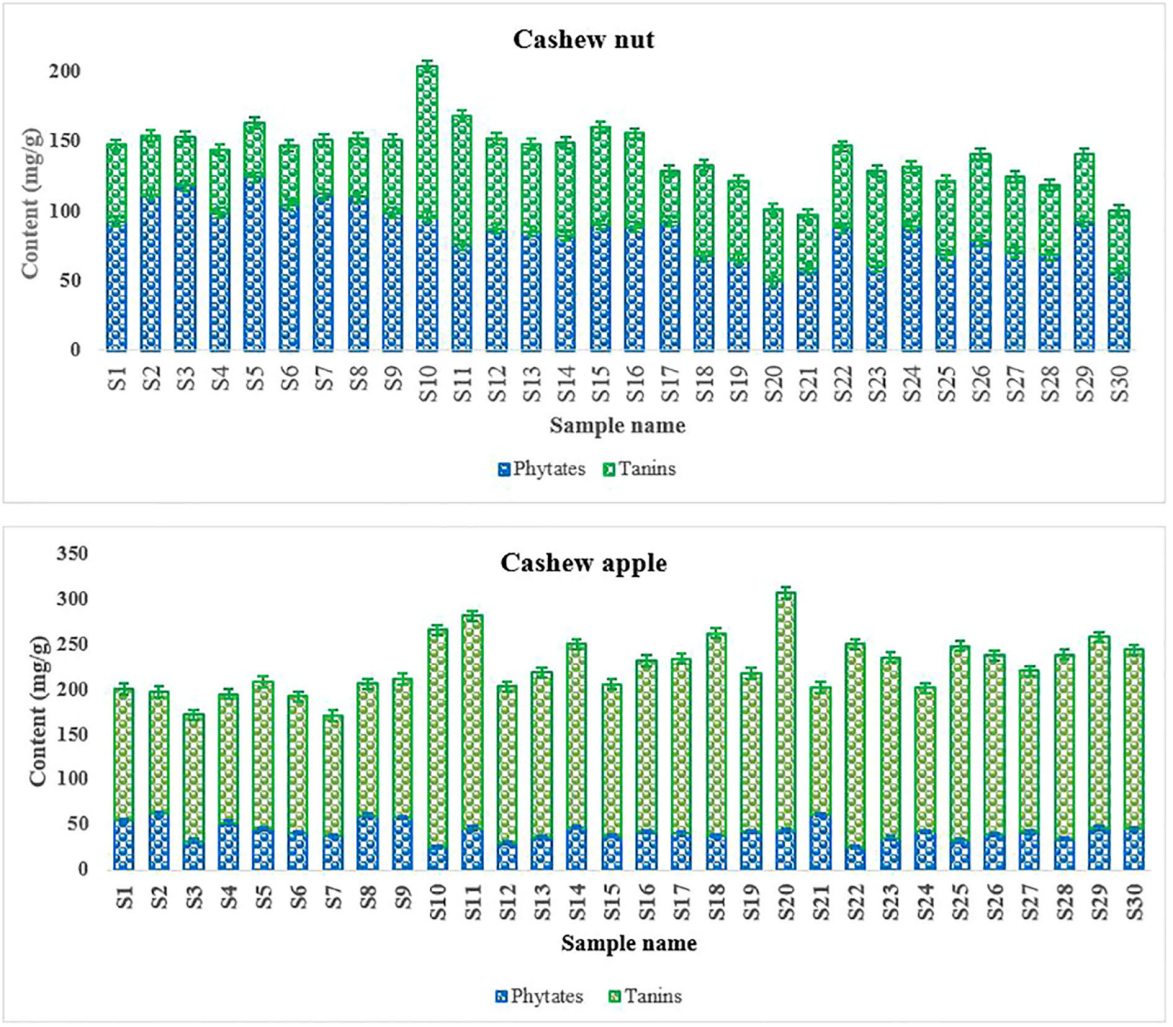
Phytates and tannins content of cashew fruits.

Hydrolysable tannins and phytates are often referred as antinutritional compounds because of their complexation with proteins/enzymes and minerals in the body, reducing their bioavailability (Amon et al., 2018, Petroski and Minich, 2020). Tannins were more abundant in apples (132,45 ± 8,4 à 224,45 ± 9,45 mg/100 g) than in kernel (35.21 ± 3.32 à 93.10 ± 7.15mg/100 g), while phytates present higher levels in kernels (49.48 ± 5.45 à 124.45 ± 9.45 mg/100 g) compared to apples (25.15 ± 3.45 à 62.21 ± 4.21 mg/100 g) (**Figure 6**). The results reveal great variations in the contents of anti-nutritional factors depending on the accessions. Indeed, the biosynthesis of these compounds can be largely influenced by biotic and abiotic stresses (Dicko et al, 2005). The abundance of tannins in apples would partly explain their astringent taste. It may not advisable for pregnant women and children to consume large quantities of foods rich in tannins (Karamać, 2009). Fortunately, hydrolysable tannins can be degraded in apples by enzymatic and thermal treatments (Al-taher, 2008). In addition, at low doses, tannins may be interesting in human health because of their antioxidant activity, inhibition of the growth of various groups of pathogenic microorganisms (Chung et al., 1998 and Adegunwa et al., 2020). Phytic acid (myoinositol hexaphosphate) is also known to reduce the absorption of certain minerals such as bivalent cations, Fe, Cu, Zn, Ca, etc. (Ogunwolu et al., 2015; Gibson et al., 2018).

Both parts of cashew fruits grown in Burkina Faso are very rich in potassium with contents ranging from 548.45mg/100 g to 630.40 mg/100 g for apples and 220.80 mg/100 g to 320.18 m/100 g (**Table 5**). These data are similar to the results of a study conducted on cashew nuts from India, Brazil, Ivory Coast, Kenya, Mozambique and Vietnam in which the average levels were 622 mg/100 g DW) (Rico et al., 2016). After potassium, sodium (425 ± 15 mg/100 g), is the most abundant mineral in cashew kernels, followed by phosphorus (402.8 ± 17.24 mg/100 g), chlorine (386.2 ± 12.4 mg/100 g), magnesium (224.6 ± 14.1 mg/100 g), iron (47.8 ± 8.2 mg/100 g) and calcium (28.9 ± 5 mg/100 g). Cashew apples, in addition to potassium, contain some minerals in interesting quantities such as phosphorus (186.2 ± 12.4 mg/100 g), magnesium (146.6 ± 12.4 mg/100 g), iron (62.5 ± 5.2 mg/100 g) and sodium (54.8 ± 7.2 mg/100 g) (**Table 5**). Elements such as copper, zinc, and manganese were found in trace amounts in both kernels and apples. Present data are in line with those found by some previous works (Msoka et al., 2017; Preethi et al. (2021), with slight differences that can be explained by the methods used or by the nature of the soil. Level of minerals in edible fruits are interesting because they are cofactors for certain proteins including metallo-enzymes, and are involved in the formation of tissues and in certain hormonal biosynthesis (Delfini et al., 2020). The results show a great variability between the cultivars concerning the content of the different minerals quantified, this is explained by the chemical composition of the soil, the fertilizers used and to a lesser extent the phytosanitary treatments that the plants undergo (Zheng et al., 2022). S means “sample collection Site” and the numbers 1 to 30 indicate the number assigned to each site as listed in **Table 1** Classification of cashew nuts on agro-morphological and nutritional characteristics

**Table 5:** Mineral content of cashew nut and apple (mg/100 g).

Cashew nut											Cashew apple									
Code	Mg	Cu	Mn	Fe	Zn	K	Na	Ca	P	Cl	Mg	Cu	Mn	Fe	Zn	K	Na	Ca	P	Cl
**S1**	20.15^c^	0.19^b^	1.04^ab^	22.15^a^	2.14^b^	301.45^a^	22.53^a^	8.24^b^	44.32^b^	2.67^a^	246.25^a^	3.96^a^	1.78^b^	4.98^a^	6.97^a^	576.45^b^	430.46^a^	51.67^a^	384.05^b^	335.50^ab^
**S2**	38.45^a^	0.24^ab^	0.92^ab^	21.30^a^	3.13^b^	298.54^a^	21.20^a^	8.23^b^	45.90^b^	2.86^a^	244.72^a^	4.02^a^	2.02^ab^	4.88^a^	7.23^a^	569.45^b^	416.32^b^	54.30^a^	402.52^b^	372.40^a^
**S3**	28.15^b^	0.21^b^	0.80^b^	15.14^b^	2.14^b^	312.50^b^	20.27^b^	8.15^b^	46.48^b^	2.45^a^	245.15^a^	4.15^a^	1.54^b^	5.60^ab^	7.15^a^	584.35^b^	427.42^ab^	55.12^a^	378.15^b^	366.15^a^
**S4**	33.94^ab^	0.16^b^	0.82^b^	17.82^b^	2.05^b^	307.54^b^	18.16^b^	8.73^b^	47.15^b^	3.12^a^	238.37^a^	4.21^a^	1.60^b^	5.24^ab^	7.26^a^	582.45^ab^	431.50^a^	52.45^a^	395.15^b^	324.78^a^
**S5**	32.15^b^	0.24^ab^	0.94 ^ab^	19.20^ab^	2.50^b^	314.32^a^	22.70^a^	8.90^b^	46.28^b^	2.87^a^	240.20^a^	3.98^a^	1.92^b^	5.37^ab^	7.68^a^	575.24 ^b^	398.45^b^	55.80^a^	364.71^b^	338.42^ab^
**S6**	29.46^b^	0.31^a^	1.20^a^	20.94 ^b^	2.84^b^	310.2^a^	20.49^a^	8.34^b^	44.98^b^	2.97^a^	228.30^ab^	3.12^a^	1.85^b^	5.62^ab^	7.45^a^	548.45^b^	397.55^b^	50.20^a^	410.34^ab^	342.42^ab^
**S7**	27.61^b^	0.19^b^	0.88^b^	22.35^a^	1.98^c^	299.25^a^	21.73^a^	8.42^b^	48.24^b^	2.38^a^	247.20^a^	3.24^a^	2.07^ab^	5.47^ab^	6.48^a^	560.40^b^	408.64^ab^	48.80^ab^	378.45^b^	346.27^ab^
**S8**	28.93^b^	0.24^ab^	1.12^a^	20.10^a^	2.24^b^	305.61^a^	22.23^a^	9.12^ab^	42.56^b^	2.84^a^	223.50^ab^	4.13^a^	1.68^b^	5.62^ab^	7.10^a^	587.45^ab^	412.61^a^	52.45^a^	369.42^b^	352.42^ab^
**S9**	24.70^bc^	0.17^b^	1.07^ab^	13.16^b^	2.32^b^	301.64^a^	20.18^a^	7.62^b^	46.23^b^	3.04^a^	198.52^b^	4.00^a^	1.90^b^	5.35^ab^	7.24^a^	602.40^ab^	402.75^ab^	49.50^ab^	395.45^b^	342.88^ab^
**S10**	32.54^b^	0.18^b^	0.87^b^	22.70^a^	1.97^c^	320.18^a^	22.04^a^	7.84^b^	44.24^b^	2.94^a^	245.48^a^	4.23^a^	2.20^ab^	5.42^ab^	6.76^a^	592.15^ab^	400.54^ab^	47.82^ab^	380.50^b^	324.80^ab^
**S11**	24.35^bc^	0.27^a^	1.16^a^	17.32^b^	3.02^b^	288.60^a^	23.24^a^	10.14^a^	49.24^a^	3.20^a^	232.50^ab^	2.96^a^	1.27^b^	5.84^ab^	6.45^a^	587.12^ab^	398.86^b^	50.14^a^	362.40^b^	362.15^a^
**S12**	22.34^c^	0.20^b^	0.78^b^	18.50^ab^	3.15^b^	288.45^a^	24.53^a^	9.28^ab^	51.42^a^	3.95^a^	220.40^ab^	3.20^a^	1.98^b^	4.76^ab^	6.02^a^	605.27^ab^	426.27^a^	52.47^a^	342.52 ^b^	356.42^ab^
**S13**	26.48^bc^	0.29^a^	0.85^b^	19.40^ab^	4.15^b^	304.42^a^	22.31^a^	13.45^a^	52.50^a^	4.02^a^	225.67^ab^	3.23^a^	2.62^a^	4.75^b^	5.86^b^	600.54^ab^	437.15^a^	48.51^ab^	398.45^b^	378.45^a^
**S14**	22.45^c^	0.26^ab^	0.90^b^	18.15^ab^	4.01^a^	275.46^a^	21.50^a^	12.37^a^	50.62^a^	3.87^a^	234.57^ab^	2.70^a^	2.04^ab^	4.86^b^	5.94^b^	586.24^b^	405.60^ab^	45.27^ab^	421.45^a^	326.80^b^
**S15**	28.45^b^	0.22^b^	0.78^b^	16.15^b^	4.17^a^	268.46^ab^	23.19^a^	10.67^ab^	54.23^a^	3.42^a^	226.40^ab^	2.48^a^	2.38^a^	4.82^b^	6.20^a^	584.15^b^	410.27^ab^	41.80^a^	418.50^a^	356.24^ab^
**S16**	30.38^b^	0.28^ab^	0.84^b^	16.16^b^	3.54^b^	279.68^a^	24.06^a^	11.48^a^	50.18^a^	4.26^a^	234.15^ab^	3.12^a^	2.47^a^	4.80^b^	6.40^a^	612.65^ab^	422.96^a^	38.94^b^	423.02^a^	328.37 ^a^
**S17**	27.94^b^	0.21^b^	0.69^c^	15.75^b^	4.02^a^	296.45^a^	25.16^a^	11.32^ab^	52.10^a^	4.23^a^	230.12^ab^	3.30^a^	3.42^a^	4.97^b^	6.47^a^	623.15^a^	402.59^ab^	40.64^b^	421.74^a^	369.20
**S18**	24.42^bc^	0.23^ab^	1.02^ab^	20.15^a^	3.18 ^b^	296.14^a^	22.98^a^	14.02^a^	51.40^a^	3.78^a^	223.40^ab^	3.24^a^	2.45^a^	5.02^b^	7.24^a^	615.45^a^	475.30^a^	41.87^ab^	411.80^a^	352.15
**S19**	26.38^bc^	0.27^a^	0.88^b^	18.73^ab^	3.49^b^	268.45^ab^	23.12^a^	10.90^ab^	49.97^a^	4.15^a^	227.50^ab^	3.50^a^	2.76^a^	4.45^a^	7.15^a^	604.87^ab^	432.15^ab^	42.15^ab^	423.20^a^	325.82
**S20**	23.81^bc^	0.30^a^	0.92^ab^	17.94^b^	2.96^b^	287.42^a^	21.96^a^	11.82^ab^	48.32^b^	3.85^a^	240.40^a^	3.40^a^	3.21^a^	4.28^b^	7.24^a^	625.48^a^	454.25^a^	50.40^a^	417.54^a^	347.42
**S21**	31.52^b^	0.32^a^	1.04^ab^	22.30^a^	2.84^b^	245.30^ab^	19.64^a^	9.27^b^	43.62^b^	3.46^a^	228.78^ab^	2.76^a^	3.02^a^	5.04^a^	7.86^a^	623.48^a^	472.15^ab^	39.95^b^	406.18^ab^	320.40
**S22**	26.83^bc^	0.24^ab^	1.16^a^	21.47^a^	2.61^b^	227.57^b^	20.14^a^	7.15^b^	45.32^b^	3.12^a^	215.60^ab^	2.73^a^	2.98^a^	5.47^a^	8.14^a^	612.05^ab^	462.40^a^	37.14^b^	403.24^ab^	318.24
**S23**	34.50^a^	0.26^ab^	1.20^a^	20.60^a^	2.82^b^	223.47^b^	19.14^a^	7.24^b^	40.24^c^	3.80^a^	207.85^b^	2.45^b^	3.23^a^	5.84^a^	7.98^a^	608.40^ab^	476.04^a^	35.21^b^	443.67^a^	321.54
**S24**	22.12^c^	0.21^b^	0.98^ab^	19.96^ab^	2.84^b^	243.80^ab^	20.32^a^	8.24^b^	41.83^b^	3.72^a^	209.40^b^	2.85^b^	3.05^a^	6.14^a^	8.21^a^	603.48^ab^	428.45^ab^	40.25^b^	435.80^a^	308.25
**S25**	28.20^bc^	0.23^ab^	1.08^ab^	18.64^ab^	2.73^b^	253.16^ab^	21.18^a^	9.01^ab^	39.94^c^	3.79^a^	212.87^b^	3.02^a^	3.11^a^	5.87^a^	8.04^a^	609.45^ab^	427.40^ab^	38.45^b^	438.54^a^	329.42
**S26**	30.48^ab^	0.28^a^	1.06^ab^	25.45^a^	2.97^b^	258.17^ab^	19.78^a^	7.86^b^	38.20^c^	3.54^a^	200.78^b^	2.78^b^	3.14^a^	6.20^a^	8.14^a^	589.40^ab^	423.48^ab^	39.70^b^	436.40^a^	314.80
**S27**	25.43^bc^	0.29^a^	1.14^a^	24.20^a^	3.40^b^	220.80^b^	18.95^a^	6.98^b^	42.20^b^	3.82^a^	218.65^ab^	2.95^a^	2.96^a^	5.73^a^	7.98^a^	596.42^ab^	456.40^a^	41.24^ab^	424.70^a^	320.70
**S28**	28.16^b^	0.27^a^	1.13^a^	23.87^a^	2.64^b^	237.84^b^	17.27^b^	7.83^b^	41.67^b^	3.42^a^	204.34^b^	2.48^b^	2.78^a^	5.98^a^	8.20^a^	618.48^a^	462.73^a^	40.60^ab^	418.74^a^	312.92
**S29**	23.72^bc^	0.30^a^	0.96^ab^	21.68^a^	3.07^b^	248.15^ab^	18.64^b^	8.15^b^	43.03^b^	4.02^a^	217.28^ab^	2.64^b^	3.32^a^	6.24^a^	8.45^a^	616.87^a^	423.84^ab^	35.68^b^	416.47^ab^	308.54
**S30**	32.75^ab^	0.25^ab^	1.22^a^	20.94^a^	2.38^b^	275.14^a^	20.64^a^	7.89^b^	41.21^b^	3.24^a^	220.10^ab^	2.76^b^	3.42^a^	6.10^a^	7.48^a^	630.40^a^	445.20^ab^	36.72^b^	434.82^a^	325.67

Superscript letters denote the statistical differences (results of the ANOVA analysis) between the samples for the parameters considered.

S means “sample collection site” and the numbers 1 to 30 indicate the number assigned to each site as listed in Table 1.

**Figure 7:**
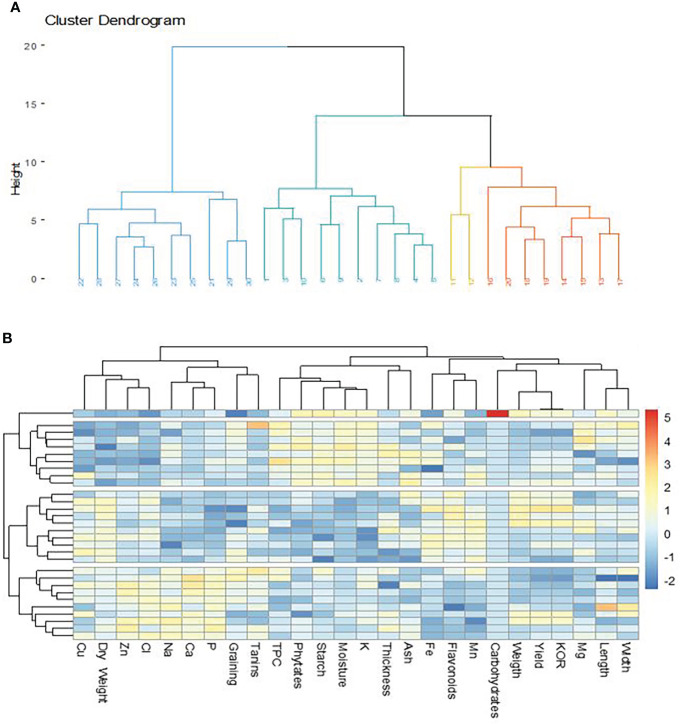
Classification of cashew nuts on agro-morphological and nutritional characteristics. **(A)** dendrogram showing the groups according to the accessions; **(B)** dendrogram function showing correlation with agro-morphological and nutritional.

**Figure 8:**
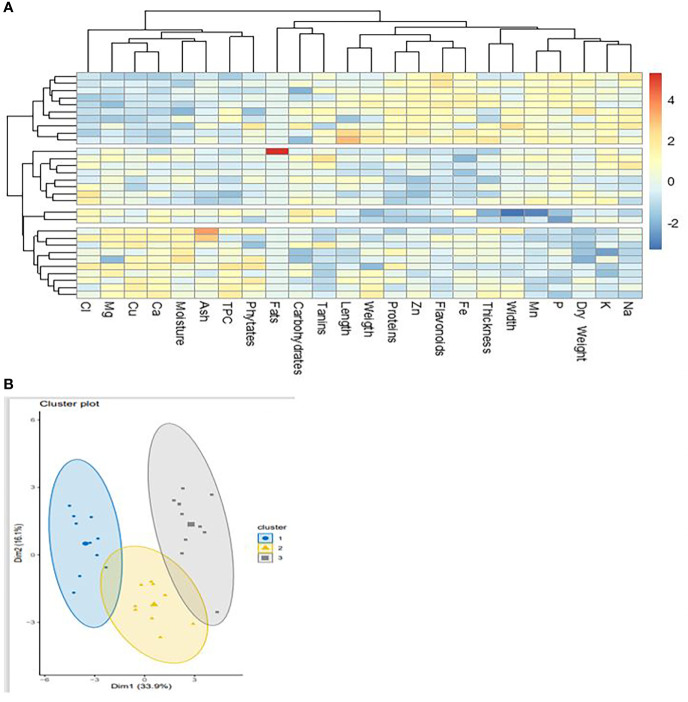
Classification of cashew apples on agro-morphological and nutritional characteristics. **(A)** dendrogram showing the groups according to the accessions; **(B)** Principal component analysis of apple samples from the 30 accessions.

Three distinct groups emerged on the dendrogram concerning the classification of cashew nuts according to their agro-morphological and nutritional characteristics (**Figure 7A**). The parameters did not show a strong correlation among groups (**Figure 7B**), which shows that cashew nuts have very similar nutritional properties and that their content is not greatly influenced by area and variety. However, the formation of three groups indicates that the accessions may be divided according to their content. This would be dependent on the area of cultivation as the three groups reflect the three regions of collection. Semporé et al. (2021a) revealed four groups based on agro-morphological characters alone in a study of 53 accessions. This difference is because this classification did not take into account the nutritional composition of the nuts. It is also observed that minerals are more correlated to group III, which is attributable to the pedology of the cultivation sites. The classification of apples also follows a similar pattern to that of walnuts, as there are also three classes (**Figure 8A**). This is usual when it is known that both parts of the fruit are linked to each other and receive the same nutrients during ripening. The nutritional parameters did not show a particular correlation among the group. This means that apples from different cultivars can be used equally in the food industry either for juice production or any other formulation. Only groups I and III are more dispersed compared to group I which seems more homogeneous (**Figure 8B**). The rate of variability between cashew samples is 50%, i.e. 33.9% and 16.1% for the two axes. Thus, the principal component analysis showed that the three groups identified do not show widely different characteristics. Indeed, the three regions concerned, which constitute the cashew nut production area in Burkina Faso, are located in the same climatic zone. The differences observed can be explained by edaphic conditions. The p-values are significant as they are around 0.0324 and the r2 around 0.62. These values show the strength of the principal component analysis.

**Figure 9:**
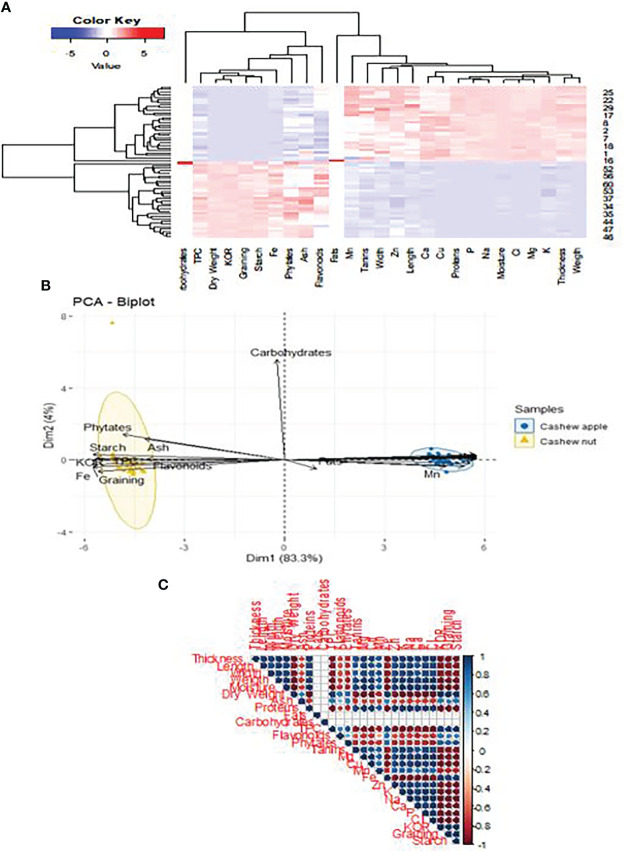
Classification of cashew apples & nuts on agro-morphological and nutritional traits. **(A)** Dendrogram function showing correlation with agro -morphological and nutritional **(B)** Dendrogram showing the groups according to the accessions and part of fruit **(C)** Correlating between different parameters.

A multivariate analysis integrating the two parts of the cashew fruit taking into account their morphological and nutritional characteristics allows to establish a clear differentiation between apples and nuts (**Figure 9B**). Parameters such as phytate, starch, flavonoid and total polyphenol content are strongly correlated with cashew nuts. In this case, the variability is quite high as it is around 87%. The values of p and r2 are respectively 0.024 and 79.14%. They support the fact that the two parts of the cashew fruit have totally different biological properties. Thus, it appears that the carbohydrate content has a very strong positive correlation with apples (**Figure 9A**). Moreover, it appears that walnuts are positively correlated with the contents of total phenolics (TPC), Fe, phytates, Flavonoids and fats. Apples would contain some contents because the principal component analysis shows a positive correlation between apple and contents of Mn, Zn, Ca, Cu, P, Mg, K (**Figure 9C**). Previous studies have confirmed that apples are very rich in sugars and minerals (Singh et al., 2019; Preethi et al., 2021). Kernels have also been proven to be among the best oilseeds (Mah et al., 2017). There are weak relationships between the agro-morphological and nutritional parameters (**Figure 9C**). However, the KOR and graining are positively correlated with the content of certain minerals such as Mg, Zn, K, Fe, Cu. This finding is very important since fruits are an integral part of the food formulas of many diets. That may allow guiding the food industries in the production of fortified foods for specific groups such as children, pregnant women, elderly people, etc.

## Conclusion

Briefly, the cashew tree is a plant whose fruit consists of two parts which are the apple and the nut. These two parts have distinct morphological characteristics. Apples contain a lot of water and are larger in size than nuts, while nuts are rich in fat and protein. The study of phytonutrients has revealed that nuts are rich in phenolic compounds but also contain high amounts of anti-nutritional factors such as phytates. Apples, although they contain very high levels of carbohydrates, are rich in tannins, which would explain their astringent taste. The most abundant minerals in cashew fruits are potassium (K), phosphorus (P), chlorine (Cl) and magnesium (Mg). They are more concentrated in apples compared to nuts. The multivariate analyses allowed to characterize three distinct groups according to morphological characteristics and nutrient contents. The mineral content was positively correlated with quality parameters such as KOR, graining. The study also established a link between certain morphological characteristics and physicochemical and nutritional traits of apples and Kernels. Such results are interesting for selecting accessions with high nutritional and economic potential.

## Data availability statement

The raw data supporting the conclusions of this article will be made available by the authors, without undue reservation.

## Author contributions

The study protocol was drafted by RD, KK, and MD. The manuscript was written by RD, KK, DB, HS, and MD. Performed the statistical analyses by RD, AS, KB, FAK, and BS. Scientific supervision of the study was provided by MD. All authors contributed to the article and approved the submitted version.

## Acknowledgments

The West African Biotechnology Network (RABIOTECH, ISP/IPICS project N° 172 600 000) is appreciated for research support and academic mobility.

## Conflict of interest

The authors declare that the research was conducted in the absence of any commercial or financial relationships that could be construed as a potential conflict of interest.

## Publisher’s note

All claims expressed in this article are solely those of the authors and do not necessarily represent those of their affiliated organizations, or those of the publisher, the editors and the reviewers. Any product that may be evaluated in this article, or claim that may be made by its manufacturer, is not guaranteed or endorsed by the publisher.
